# Developing a Model for National Health Information Governance Program in Iran

**DOI:** 10.25122/jml-2020-0036

**Published:** 2020

**Authors:** Fatemeh Rouzbahani, Farkhondeh Asadi, Reza Rabiei, Hamid Moghaddasi, Hassan Emami

**Affiliations:** 1. Department of Health Information Technology and Management, School of Allied Medical Sciences,Shahid Beheshti University of Medical Sciences, Tehran, Iran

**Keywords:** Information governance, health, Iran

## Abstract

With regard to the importance of health Information Governance (IG) programs in improving the quality and reducing the cost of healthcare services and the lack of a coherent health IG program in Iran’s health system, this study aimed to develop a model for national health information governance program in Iran.

The present research was an applied, cross-sectional descriptive study that was done in three steps, including literature review, development of a model for national health IG program in Iran, and model validation. In the third step, we used a questioner to validate the model through the Delphi method. Data analysis was done by descriptive statistics.

The model for the national IG program in Iran was developed in 3 main sections consisting of 13 components, 12 principles, natural and judicial authorities of the health IG program, and their job description. Findings from the validation of the initial model showed that most experts (93%) confirmed the components and sub-components, principles, and natural and legal bodies supervising the national health IG program and their job description in the proposed model.

Considering the structure of the Iranian health system, it was recommended to establish a health IG council in the Ministry of Health and Medical Education in order to develop guidelines and give advice to health care providers. Based on the proposed model, directors and staff of different departments of health care centers, especially those involved in health IG, are also responsible for the better implementation of the national health IG program.

## Introduction

The health system of any country is organized and developed based on that country’s needs and resources. A desirable health system is expected to provide high-quality services to the community at any time and place [1]. Although different countries may provide services of different qualities to their citizens, any health system is expected to provide quality care for individuals, maintain community health, reduce per capita healthcare costs, and adopt the best policies and decisions based on valid information [2, 3]. In other words, the reflection of quality improvement approaches relies heavily on the data and information related to measures and evidence for positive changes [2]. These objectives can be achieved by incorporating information, as a strategic resource, into decisions and plans at different health system levels [5]. Information governance (IG) is a comprehensive organizational tool used in a health system to efficiently manage the information and support organizational strategy and operational, legal, safety, and environmental requirements [6, 7]. In fact, it is a strategic framework consisting of the standards, processes, roles, and criteria used by organizations and individuals for creating, organizing, securing, preserving, using, and eliminating information in line with organizational goals [8].

The current challenges of the health industry, such as the growing and diverse volume of data and information, extensive use of health information, interoperability of information systems, and the need for data exchange and sharing, have highlighted the need for the development and adoption of a health IG system [8].

Despite the high potential of health care information and the need to improve the quality of care, the management of paper or electronic medical records has not yet been properly addressed. As a result, these data are distributed in separate repositories in different formats that prevent data sharing [9]. Moreover, there are technical problems with interaction and security as well as ethical, legal, and regulatory requirements that prevent uninterrupted data sharing; however, they are designed to protect data privacy [10]. The high volume of health care data and the need to integrate them strongly necessitate the application of the IG approach.

Developed countries have found out the significance of health IG. For example, the Health and Social Care Information Centre (HSCIC) in the United Kingdom is responsible for health IG through a self-assessment process [11]. This process is done using a tool consisting of 6 main requirements: IG manager, data confidentiality and protection assurance, information security assurance, clinical information assurance, secondary use assurance, and corporate information assurance. Depending on the type of organization, each requirement may include a different number of items [12]. According to Willison et al., the implementation of an IG program is influenced by eight principles, including transparency, accountability, follow the rule of law, integrity, participation and inclusiveness, impartiality and independence, effectiveness, efficiency and responsiveness, and reflexivity and continuous quality improvement [13].

In the United States, the health IG program is considered a strategic necessity in the healthcare industry and consists of information access, information security and confidentiality, integrity and quality of information, and information design and collection [14].

The health IG program in Canada is a combination of legal, ethical, and regulatory requirements for the collection, application, or disclosure of personal health information [15]. This program also includes records and information management, data privacy, information security, and electronic discovery [16].

Given the applications and importance of health IG programs in improving the quality and reducing the cost of healthcare services and the lack of a coherent health IG program in Iran’s health system, it is necessary to develop a national health IG model for Iran’s health system by taking into account the components and principles of health IG as well as the organizations and individuals responsible for its implementation.

## Material and Methods

The present research was an applied, cross-sectional descriptive study conducted in three stages.

### Literature review

Because of the more significant history and experience of countries such as the United States, Canada, and the United Kingdom in the implementation of health IG programs [17, 18], the relevant papers published by these countries from 2000 to 2017 were searched on PubMed, Science Direct, Scopus, and ProQuest. The keywords used for the search included “information governance”, “information governance program”, and “impacts of information governance on healthcare”. In addition, part of the data was collected from reputable websites such as the American Health Information Management Association (AHIMA) website.

### Development of a model for the national health IG program in Iran

To develop a national health IG model, the health IG programs implemented in selected countries were reviewed. Then, an initial model was developed based on similarities and differences of countries in relation to components, principles, and authorities of the health IG program and organizational structure of Iran’s health system.

### Model validation

The proposed model was validated in two steps using the Delphi method. To this end, a researcher-made questionnaire that consisted of three sections of components, principles, authorities of a health IG program was developed. The items were scored based on a 3-point Likert scale (agree, agree to some extent, and disagree). In addition, the questionnaire was designed in a way that the respondents could provide their suggestions. To measure the validity of the questionnaire based on content validity, the questionnaire was sent to 5 experts in health information management in order to elicit their views and comments. The reliability of the questionnaire was assessed using Cronbach’s alpha. Accordingly, Cronbach’s alpha coefficient for this questionnaire was 86%, which indicates the high reliability of the questionnaire results. Once the questionnaire validity and reliability were confirmed, it was given to 10 experts in health information management, 2 experts in information technology management, and 3 faculty members of Medical Informatics from Tehran Medical University and Shahid Beheshti University of Medical Sciences in order to elicit their views and comments on the main components of the proposed model for the national health IG program. A 75% coefficient of agreement was considered the criterion for model acceptance. Then, experts’ views and comments were applied to the model. In the second step of the Delphi method, a panel of experts attended by 7 health IT management specialists finalized the proposed model (it is noteworthy that all panel members were selected from individuals who had years of experience in the implementation of information quality processes in the health system). The data related to the final model approval by experts were statistically analyzed using descriptive statistics (frequency and percentage).

## Results

The findings of the literature review indicated that the common objective of health IG programs in all countries is to improve the quality of patient care and public health, reduce medical costs, and implement mechanisms that ensure the quality, access, and security of information throughout the healthcare system. The model for the national IG program in Iran was developed in 3 main sections consisting of 13 components, 12 principles, natural and judicial authorities of the health IG program, and their job description (Tables 1, 2, and 3).

**Table 1:** Components and sub-components of the proposed model.

Components	Sub-components
**Rules and regulations**	Information and data confidentiality rules, data security rules, information and cyberspace, information management rules
**Policies**	Information and data confidentiality policy, data and information security policy, information and documents management policy
**Standards**	Health IG management standard, information and data confidentiality standard, data, and information security standard, information, and documents management standard, data and information quality control standard
**Information management**	Information design, creation, and gain, information analysis, information access and application, information dissemination, information storage, information disposal, information quality management, coding based on international coding books such as ICD (International Classification of Diseases)
**Documents management**	Documents design, creation, access, and application of documents, documents dissemination, documents storage, documents disposal, documents quality management
**Data Governance**	Definition of data elements, determination of data structure and architecture, data modeling, improvement of clinical documentation, data quality control and management, big data management, metadata management, data dictionary management
**Information technology (IT) Management**	Alignment of IT strategies with organization strategies, IT processes management and direction, development of an integrated approach to the selection, evaluation, and use of IT, application of technologies to manage and control information and documents, IT resources management such as information systems, hardware and software, network and database communications, financial and human resources, and data and information
**IT Governance**	IT processes management and direction, development of an integrated approach to the selection, evaluation, and use of IT, application of technologies to manage and control information and documents, monitoring IT resources such as information systems, hardware and software, network and database communications, financial and human resources, and data and information
**Risk Management**	Identification of the events threatening data, information and cyberspace, prevention and control of unwanted events such as distortion, data and information theft, and violation of the security and privacy of data, information, and cyberspace, analysis and evaluation of threats and damages, risk management through setting and enforcing data and information access and protection policies, development and implementation of post-disaster data recovery programs
**Change Management**	Planning for development of new structures, processes, and technologies, implementation of changes or reforms, occupational development and enrichment, change control,evaluation and fixation
**Compliance Management**	Identification of internal and external laws, policies and standards, compliance with internal and external laws, regulations and standards, responding to legal requests, evaluations, and audits
**Human Resource Management**	Identification, selection, and recruitment of employees, job design and analysis and assignment of roles and responsibilities, staff training and justification, compensation management,performance evaluation
**Program monitoring and auditing**	Definition and formulation of necessary criteria, criteria-based evaluation at specified intervals, provision of feedback for decision-making and program improvement

**Table 2:** Principles of the proposed model.

**Accountability**	A legally responsible and accountable executive member of senior management shall control the IG program and delegate the responsibilities.
**Transparency**	Processes and activities related to an organization's IG program shall be developed in a clear manner. These documents shall be available to the staff and other stakeholders with regard to legal or regulatory restrictions and in line with business needs.
**Integrity**	The IG program shall be developed in a way that ensures information reliability during the process of information creation, storage, and presentation.
**Compliance**	The IG program shall be in compliance with organizational laws, regulations, standards, and policies.
**Data and information quality**	Accurate, up-to-date, relevant, and sufficient data and information shall be collected, stored, and retained.
**Data and information protection**	The IG program shall ensure an acceptable level of protection against infringement, corruption, and lack of information that is private, confidential, classified, and necessary for the continued operation of the organization. Data protection and attention to the security and confidentiality of data and information shall be regardless of the media type when creating, presenting, and even removing information.
**Data and information accessibility**	An organization shall retain information in a way that ensures timely, accurate and efficient data recovery. In order for effective management of information accessibility, an organization shall eliminate additional and obsolete data and information at regular intervals.
**Data and information retention**	Considering the legal, regulatory, financial, and operational requirements, an organization shall retain its information for an appropriate period of time.
**Data and information elimination**	An organization shall adhere to organizational rules and policies to safely and appropriately eliminate unnecessary information. At the end of the information retention period, additional and unnecessary information shall be eliminated.
**Legality of data processing**	The data shall be processed fairly and in accordance with the legal objectives and policies of the organization.
**Monitoring and auditing**	Compliance with policies and standards, staff performance, access to and use of information and other informational media, the use of technologies such as Document Analytics shall be monitored.
**Continuous improvement**	The IG program shall be reviewed regularly and periodically in order to be modified and improved based on defects, workplace changes, new technologies, and organizational strategies.

**Table 3:** Natural and legal bodies supervising the national health IG program and their job description.

Authority	Job description
**Minister of Health and Medical Education at the national level (Board of directors/head in health care organizations)**	Responsibility, accountability, and provision of all necessary resources related to the health IG program
**Director of the national health IG program (in health care organizations)**	Coordination, development, and implementation of the program, resource identification and proper use, managerial responsibilities, identification of information management rules and regulations, development and implementation of a monitoring system,identification of workforce training needs
**Information governance and management advisor (in health care organizations)**	Consultation services for the program design and implementation, evaluation and improvement of existing policies, and design, documentation, and adoption of the best practices
**Director of information security and confidentiality (in health care organizations)**	Provision of counseling and relevant content, data privacy protection, ensuring the legal, ethical and proper use and retention of information, information accessibility monitoring and audit, coordination of relevant programs
**Director of risk management (in health care organizations)**	Promotion of data protection culture, provision of important points for managing risks and events threatening all kinds of data and information
**Data steward (in health care organizations)**	Development of policies and methods, organizing and implementing policies and practices for monitoring published reports, addressing any issue related to data integrity and quality, communication with employees, identification of the existing source of information and data, management of different data, security and privacy of different data
**Executive teams/working groups of the health IG program (in health care organizations)**	Identification of new opportunities, promotion of the culture of IG, monitoring related policies, processes and problems, security and confidentiality, integrity and quality of information, information design and gain, document content and information management, information analysis, application, and exchange
**Directors of different departments (in health care organizations) **	Ensuring the implementation of policies and procedures, application of a monitoring program, adherence to principles and all relevant laws, standards, policies, and procedures, subordinates’ awareness of the rules, policies, standards, and responsibilities in relation to the health IG program
**Director of health information management (in health care organizations) **	Collaboration in design, implementation, and guidance, strategic planning, monitoring the organization’s compliance with the relevant policies, identification of threats and opportunities, evaluation of positive effects of the health IG program
**Staff of health care organizations**	Safe creation, application, processing, retention, and deletion of information and data, proper use of IT systems, participation in relevant training courses
**Steering Committee of the health IG program**	Development of policies, standards, evaluation tools, and reporting tools
**IG Council (Ministry of Health and Medical Education)**	Valid source for advice and guidance on program implementation, preparation of a collection of important information related to data privacy, security, and management, development of IG networks (strategic information governance networks, abbreviated as SIGNs) in universities of medical sciences
**Health IG Committee (in health care organizations)**	Development of an appropriate framework, strategy, and working plan, coordination of staff activities, monitoring and auditing the IG program activities, support for the executive team, discussion on lessons learned from the health IG events
**Health Information Management Association**	Provision of educational information and resources, organizing conferences and workshops related to the health IG program

Table 1 showed the components and sub-components of the proposed health IG model for Iran, which was conducted at the second step of the study.

Table 1. Components and sub-components of the proposed model.

Table 2 showed the principles of the proposed model for the health IG program for Iran, which was classified in 12 main categories.

Table 3 showed the natural and legal bodies supervising of the national health IG program for Iran and their job description.

### Findings of the first step of the Delphi method

Findings from the validation of the initial model showed that most experts (93%) confirmed the components and sub-components, principles, and natural and legal bodies supervising the national health IG program and their job description in the proposed model. However, it was recommended to merge “information management” and “documents management” into one component entitled “information and documents management” and also merge “IT management” and “IT supervision” into one category entitled “IT management and supervision”. In addition, it was emphasized that special attention should be paid to policies and standards related to data, information, and cyberspace security. Table 4 presents a summary of experts’ views on the national health IG program.

**Table 4:** Overall views of experts on the national health IG program.

Experts’ views The national health IG program	Agree		Agree		Agree		Recommendations
	Number	Percentage	Number	Percentage	Number	Percentage	
**Components and subcomponents**	13	86.7	0	0	2	13.2	• Merging “information management” and “documents management” into one category • Including information/data security policies and standards in the main components • Merging “IT management” and “IT supervision” into one category
**Principles**	15	100	0	0	0	0	
**Natural and legal bodies**	14	93.2	1	0.7	0	0	

### Findings of the second step of the Delphi method

**Figure 1:**
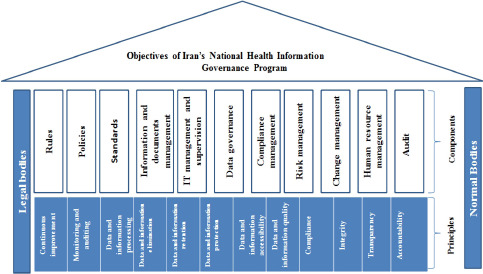
Iran’s National Health Information Governance Program (the final model).

In the second step, recommendations proposed in the previous step were applied to the model. Then, the modified model was sent to the expert panel. The model was reviewed several times and finalized by this panel. Figure 1 and Table 5 present the final national health IG program.

**Table 5:** Natural and legal bodies of Iran’s National Health Information Governance Program.

Minister of Health and Medical Education at the national level
(Board of directors/head in health care organizations)
Director of the national health IG program (in health care organizations)
Information governance and management advisor (in health care organizations)
Director of information security and confidentiality (in health care organizations)
Director of risk management (in health care organizations)
Data steward (in health care organizations)
Steering Committee of the health IG program
IG Council (Ministry of Health and Medical Education)
Health IG Committee (in health centers)
Health Information Management Association

## Discussion

The common objective of health IG programs in all countries is to improve the quality of patient care and public health, reduce medical costs, and implement mechanisms to ensure the quality, access, and security of information throughout the health system [19]. The proposed model for Iran’s Health Information Governance Program consisted of the following 11 components: rules and regulations, policies, standards, information and documents management, data governance, IT management and supervision, risk management, change management, compliance management, human resource management, and monitoring and auditing. In addition, the model included these 12 principles: accountability, transparency, integrity, compliance, data and information quality, data and information protection, information accessibility, data and information retention, data and information elimination, the legality of data processing, monitoring and auditing, and continuous improvement. Hence, the proposed model included all components of IG in health and non-health areas. For the sake of comprehensiveness, data protection rules (principles of data and information quality and processing), extracted from major health IG rules in the United Kingdom, were included in the model. In addition, there was an emphasis on principles of monitoring and auditing and continuous improvement cited in the literature on IG in non-health areas [20]. Winter and Davidson stated that health data are monitored by various stakeholders, ranging from healthcare providers to IT companies. Data governance is, therefore, defined as a system of legal decision-making and auditing for information processes. These generally accepted models describe who can use what information and practices and when, how, and under what conditions to use them [1, 21]. The present study fully addressed the natural and legal bodies, components, and principles of the IG program.

In relation to the authorities (supervisory bodies) of the health IG program, given the organizational structure of the Iranian health care system and findings from a comparative study on selected countries, the Minister of Health and Medical Education was appointed as the head of the health GI program to secure and provide the resources needed to establish and execute the program. In the United Kingdom, the Department of Health and Social Care is one of the main authorities of the health IG program [17, 20]. It was also recommended to establish a steering committee of health IG in the Ministry of Health and Medical Education to develop the necessary policies and standards. The same committee was also established in health care organizations in the US and the UK for this purpose.

Ford et al. proposed a new approach to the health IG, which considered data owners, the public and legislators, the research team, and the translatability of health knowledge. In addition, the balance between privacy and social benefits when using data was considered in this approach [22].

Considering the structure of the Iranian health system, it was recommended to establish a health IG council in the Ministry of Health and Medical Education in order to develop guidelines and give advice to health care providers. Since health information management associations play an active role in the studied countries, the Iranian Scientific Association of Health Information Management can contribute to training courses and workshops on the development and implementation of the health IG program. Similar to the countries studied, the highest authority in Iranian health care organizations (board of directors/head) is responsible for supporting the national health IG program. Based on the findings from the countries studied in this paper, other roles such as “Information governance and management advisor”, “Director of information security and confidentiality”, “Director of risk management”, and “Data steward” were also included in the proposed model. Based on the proposed model, directors and staff of different departments of health care centers, especially those involved in health IG, are also responsible for the better implementation of the national health IG program.

## Conclusion

Given the importance of clinical governance programs in reducing costs and improving the quality of health services in all countries, having a proper model for implementing clinical governance is an undeniable necessity.

## Conflict of Interest

The authors declare that there is no conflict of interest.
